# Dignity of informal caregivers of migrant patients in the last phase of life: a qualitative study

**DOI:** 10.1186/s12904-021-00721-6

**Published:** 2021-02-04

**Authors:** X. de Voogd, D. L. Willems, M. Torensma, B. D. Onwuteaka-Philipsen, J. L. Suurmond

**Affiliations:** 1 Department of Public and Occupational Health, Amsterdam UMC, University of Amsterdam, Meibergdreef 9, 1105 AZ Amsterdam, Netherlands; 2 Amsterdam Public Health Research Institute, Van Boechorstraat 7, Postbus 7057, 1007 MB Amsterdam, Netherlands; 3 Department of Ethics, Law and Humanities, Amsterdam UMC, University of Amsterdam, Meibergdreef 9, 1105 AZ Amsterdam, Netherlands; 4 Expertise Centre for Palliative Care, Amsterdam UMC, Boelelaan 1117, Postbus 7057, 1007 MB Amsterdam, Netherlands

**Keywords:** Caregivers, Dignity, Caregiver needs, Palliative care, Migrants, End-of-life care, Qualitative research

## Abstract

**Background:**

A key aim of palliative care is to improve the quality of life of patients and their families. To help ensure quality of life for the families of patients with migrant backgrounds, this study sought insights into the dignity of informal caregivers in migrant communities. This could improve understanding of family-centered care for migrant patients.

**Methods:**

Twenty semi-structured interviews with informal caregivers of Turkish, Moroccan, or Surinamese background living in the Netherlands were analyzed thematically.

**Results:**

The dignity of the patient and that of their informal caregivers were found to be strongly interrelated. Most important for the dignity of caregivers was ensuring good care for their patients and preserving the patients’ dignity. Ensuring good care involved advocating for good and dignified care and for satisfaction of a patient’s wishes. For many informal caregivers, it also included delivering care to the patient by themselves or together with other family members, despite having to give up part of their own lives. Providing care themselves was part of maintaining a good relationship with the patient; the care was to cater to the patient’s preferences and help preserve the patient’s dignity, and it could be accompanied by valuable aspects such as times for good conversations. Positive interaction between an informal caregiver and a patient positively influenced the informal caregiver’s dignity. Informal caregiver and patient dignity were often compromised simultaneously; when informal caregivers felt healthcare professionals were undermining a patient’s dignity, their own dignity suffered. According to informal caregivers, healthcare professionals can help them preserve dignity by taking seriously their advice about the patient, keeping them informed about the prognosis of the disease and of the patient, and dealing respectfully with differences in values at the end of life.

**Conclusion:**

The dignity of migrant patients’ informal caregivers in the last phase of a patient’s life is closely entwined with ensuring good care and dignity for the patient. Healthcare professionals can strengthen the dignity of informal caregivers by supporting their caregiving role.

**Supplementary Information:**

The online version contains supplementary material available at 10.1186/s12904-021-00721-6.

## Background

Different meanings of dignity can be found in history. One of these is *personal dignity*, the subjective personal sense of dignity, and this is the focus of this study. Personal dignity has been studied extensively among palliative care patients [1, 2]. The research shows that dignity consists of an personal subjective valuing of oneself, and of valuing oneself within reciprocal relationships with others. Dignity is closely related to a patient’s self-esteem [3, 4]. Dignity consists of an internal aspect, which is one’s personal, subjective valuing of oneself, and an external aspect, which is the valuing of oneself by others. Dignity can be compromised by physical and mental changes due to illness, as well as by actions of others, including relatives or healthcare professionals [3, 5]. Maintaining dignity is therefore a key aspect of quality of life. Elements integral to preserving dignity in palliative patients are maintaining their autonomy, upholding their own identity, asserting a social role, having meaningful relationships with others, and not becoming a burden to others [3, 4, 6]. In addition, studies among migrant patients in the Netherlands [7], and also of patients in China and Taiwan, have found that being cared for by family as well as spiritual surrender (for instance to God or Allah) are additional pertinent aspects of dignity in the last phase of life [8, 9]. The WHO definition of palliative care emphasizes that good palliative care entails improvement in a patient’s quality of life and also in a patient’s support system [10]. Since family often becomes highly involved in care for a patient, the dignity of close relatives becomes crucial alongside the dignity of the patient. Healthcare professionals have a significant role to play both in maintaining a patient’s dignity and in facilitating family-centered care [5, 11, 12].

In the Netherlands, informal caregivers of patients of non-Western migrant background are generally more closely involved than ethnic Dutch patients’ caregivers when it comes to caring for chronically ill patients in their last phase of life [13–16]. In comparison with ethnic Dutch patients, more migrant patients aged 55 or older who suffer from one or more chronic diseases receive family care [13]. Expectations that relatives will care for them are stronger among migrant patients [13, 14], and care by relatives helps to preserve patients’ dignity, as we found in our recent study of migrant patients in the palliative stage [7].

Previous studies have not, to our knowledge, investigated the dignity of informal caregivers, but doing so can provide valuable insights for healthcare professionals on how to support informal caregivers’ dignity and hence their quality of life. The aim of our study is therefore to gain understanding of how migrant patients’ informal caregivers experience their own dignity during the final phase of a patients’ life.

## Methods

### Design

This study about the dignity of informal caregivers is part of a larger qualitative study on the dignity of migrant patients in their last phase of life, which includes interviews with patients and their informal caregivers. In the Netherlands, migrants are defined as persons who were born outside the Netherlands or who have a parent born outside the Netherlands [17]. The term “migrants” is not officially used; the term “persons with a migrant background” is preferred. In this article we use the term “migrant,” because it is internationally better known and generally used in research about ethnic differences.

Our research focused on informal caregivers of Turkish, Moroccan, or Surinamese background, reflecting the three largest migrant groups in the Netherlands. The Surinamese community consists of two main ethnic groups, the African Surinamese and the South-Asian Surinamese; we included informal caregivers from both groups. We collaborated with migrant network organizations, which advised us during all stages of the study and helped with recruitment. We engaged two ethnically matched bilingual interviewers of Turkish and Moroccan background, one with experience in quantitative research in palliative care, the other in spiritual counseling. We provided them with interview training before the study started. As most Surinamese migrants in the Netherlands already had Dutch as their mother tongue, given the colonial history, we did not need a bilingual interviewer of Surinamese background.

#### Participants

We used purposive sampling—selecting participants based on the specific qualities needed to carry out the study [18]—in order to reach informal caregivers who were primarily tasked with caring for a patient in the last phase of life. We took care to reach sufficient informal caregivers of each ethnic background who were diverse in age and who were attending patients with various diseases. Our inclusion criterion was caregiving to patients who were in the palliative stage and/or had a life-threatening disease. We established the palliative stage on the basis of the three trajectories described by Murray and colleagues [19] in relation to type of disease and duration: (1) short period of evident decline, typically cancer; (2) long-term limitations with intermittent serious episodes, often heart failure or COPD; and (3) prolonged dwindling, often dementia or generalized frailty. Depending on trajectory and disease, the included patients could be in the last 3 months to 8 years of their lives.

We recruited informal caregivers through nursing homes with culture-sensitive units and/or multicultural populations, through a general practitioner, and through our migrant network partners. Culture-sensitive units are sections serving patients with similar countries of birth and having an eye for specific cultural or religious needs, such as a preferred cultural furnishing of rooms. We recruited in two hospitals through the palliative team and the oncology, lung diseases, and neurology departments. Nurses, physicians, Islamic spiritual counselors, and key informants of these institutions and networks informed us about patients who were in a palliative stage, based on the illness trajectories [19] we had discussed with them, and they gave such patients and their informal caregivers a language-matched information letter. After a patient and/or informal caregiver provided consent to the recruiter, we approached them in person or by telephone, informing them that the interview would be about feelings of dignity in their current situation and in the future. We avoided terms such as “last phase of life,” “palliative care,” and “incurable disease,” because our migrant network partners viewed such words as daunting for patients. The interviews were carried out at the participant’s home or nursing home in different interview configurations, depending on the patient’s preferences and abilities: patient and informal caregiver together, or separately, or informal caregiver only. Table 1 shows characteristics of the informal caregivers and the interview settings.

**Table 1:** Characteristics of participants

	Age	Highest level of education	Relationship with the patient	Religion	Country of birth informal caregiver	Country of birth patient	Care setting patient	Disease / Syndrome patient	Interview setting	Gives daily care themselves
1	56–60	Primary school	Husband	Islam	Turkey	Turkey	At home	Paralysis due to cerebral infarction	Relative and patient together	Yes, completely, together with children
2	51–55	Inter-mediate professional education	Daughter-in-law	Islam	Turkey	Turkey	Nursing home	Muscle disease	Relatives and patient together	Yes, together with care professionals
3	51–55	Secondary school	Son	Islam	Turkey	Turkey	Nursing home	Muscle disease	Relatives and patient together	Helps his wife (respondent #2)
4	46–50	Inter-mediate professional education	Daughter	Islam	Turkey	Turkey	Nursing home	Dementia	Relative and patient together	No, but had done it before
5	66–70	Secondary school	Husband	Islam	Turkey	Turkey	Nursing home	Parkinson	Relative and patient together	No, but had done it before
6	50–55	Inter-mediate professional education	Daughter	Islam	Turkey	Turkey	Home near nursing home	Parkinson and dementia	Relative only	Yes, together with care professionals
7	50–55	Secondary school	Daughter	Islam	Turkey	Turkey	Nursing home	(Vascular) Dementia, Diabetes	Relative only	No, but had done it before.
8	70–75	Secondary school	Wife	Islam	Turkey	Turkey	At home	Cancer, Diabetes	Relative of deceased patient	Yes, completely for daily care
9	46–50	Secondary school	Daughter	Islam	Turkey	Turkey	At home and hospice	Cancer, Kidney failure	Relative of deceased patient	Yes, completely
10	41–45	Inter-mediate professional education	Wife	Islam	Morocco	Morocco	At home	Cancer	Relative and patient together	No, but had done it before together with other family members
11	36–40	Higher professional education	Daughter	Islam	Netherlands	Morocco	Nursing home	Paralysis due to cerebral haemorrhage	Relative only	Yes, completely
12	41–45	Unknown	Daughter	Islam	Netherlands	Morocco	At home	Cancer	Relative only	Yes, completely
13	46–50	University	Daughter	Islam	Morocco	Morocco	At home	Cancer	Relative of deceased patient	Yes, completely
14	61–65	University	Daughter	Evangelical Brotherhood Church	Suriname	Suriname	At home	Heart disease	Relative and patient seperately	No, not needed yet but would do it
15	Unknown	Unknown	Daughter	None	Unknown	Suriname	Nursing home	Dementia	Relative and patient together	No, but had done it before together with other family members
16	41–45	Inter-mediate professional education	Daughter	None	Netherlands	Suriname	Nursing home	Dementia	Relative and patient together	No
17	56–60	University	Son	None	Netherlands	Suriname	Nursing home	Dementia	Relative and patient together	No
18	61–65	Secondary school	Daughter	None	Suriname	Suriname	Nursing home	Dementia	Relative only	No
19	Unknown	Unknown	Daughter	Unknown	Unknown	Suriname	Nursing home	Paralysis due to cerebral infarction	Relative only	No, but assists care professionals
20	Unknown	University	Daughter	Hinduism	Suriname	Suriname	At home	Dementia	Relative of deceased patient	Yes, completely

#### Data collection

We conducted interviews between December 2017 and June 2018. Interviews were carried out according to respondents’ language preferences, either in Dutch, Turkish, Berber, and/or Arabic. XV interviewed all Surinamese, four Moroccan, and one Turkish informal caregiver in Dutch. The bilingual interviewers conducted the remaining interviews; XV verified that their first two interviews were performed correctly and were in-depth.

The interview guide was based on key concepts in the literature about dignity [2–5, 8, 9] and about palliative care and culture [15, 16], which had also been addressed by key informants with whom we carried out preliminary interviews. Key informants were involved in our study from the start and were Islamic spiritual counselors, care consultants, or social workers with Turkish, Moroccan, or Surinamese backgrounds. The interview guide covered three main topics: dignity of the informal caregiver, what caregivers did to preserve a patient’s dignity, and what constitutes dignity for the patient in question. See Additional file 1 for the interview guide. Dignity was translated as *waardigheid* in Dutch, *haysiyet*, *değerli and değerlilik* in Turkish, and *karama* and *qima* in Arabic. Some participants did not understand the word “dignity” immediately or how it might relate to their situation. We believe this was not because they were unfamiliar with the notion of dignity in their culture, but because they had difficulty translating that abstract concept into their daily lives. This was confirmed by both the bilingual interviewers and the key informants, who assured us that the notion of dignity exists in their language and culture. When we used other words, such as feeling “worthy,” “appreciated,” “valued,” or “small” as synonyms for “dignified” or “undignified,” all participants were eventually able to reflect on dignity. The interviews lasted from 45 to 75 min.

Interviews were audio-recorded and then simultaneously transcribed and translated. Data collection was continued until data saturation of the main themes was reached.

#### Data analysis

We used thematic content analysis, which encompasses familiarization, identifying a thematic framework, indexing, charting, mapping, and interpretation [20]. A phenomenological perspective was taken [21]. First, we coded what relatives explicitly regarded as important for their dignity, and, in order to deepen those conceptions, we subsequently coded other parts of the interviews in which those aspects played a role. Examples are the paragraphs about “motives for the importance of family care” and “interaction of informal caregivers with the patient and significant others” which describe why a caring role is important to informal caregivers and what aspects within their interactions they found meaningful for being a good caregiver. To guarantee the rigor of the study [22], XV, JS, and the two other interviewers discussed four interview transcripts. XV and MT coded two interviews separately and compared codes. XV then developed a coding tree and coded the rest of the transcripts, adding new codes where necessary. Codes were then united into overarching themes. For example, the code “dignified behavior” was connected to the theme “interaction with the patient and significant others.” The themes, codes, and descriptions of the main findings were discussed and confirmed among XV, JS, and DW.

#### Ethical considerations

Under the Dutch Medical Research Involving Human Subjects Act [23], this study did not require approval by an ethics committee, and that was confirmed in writing by the Medical Ethics Committee of the Amsterdam Academic Medical Centre (AMC). We adhered to the ethical principles for medical research involving human subjects laid down in the Declaration of Helsinki adopted by the World Medical Association [24]. We informed participants of the aims of the study and their legal rights. Written informed consent was obtained before or directly after the interviews, and we used codes to anonymize participant information.

## Results

We interviewed 20 informal caregivers, nine of whom had a Turkish background, four a Moroccan background, three an African Surinamese background, and four a South-Asian Surinamese background. Most of the caregivers were women (16), and many were responsible for a large part of the care for their loved one, either continuing at the time of the interview (7) or in the past before the patient was admitted to a nursing home (4). All of the male respondents who were taking care of their loved ones themselves (2) were partners of the patient; the interviewed sons were not providing care themselves but were the primary contact for the patient care.

The following factors were vital to the dignity of informal caregivers: (1) dignity as providing good care, (2) the dignity of the patient, (3) their interaction with the patient and significant others, and (4) the acknowledgment and support of their caretaking role by care professionals. Caregivers also reported that the caregiving itself was part of their dignity despite having given up part of their lives, and they described several relational motives for giving care to the patient.

### Dignity as providing good care

For all informal caregivers, the provision of good care for their loved one was a prominent aspect of their own dignity. Thus, for many, ensuring that the patient received good and dignified care from healthcare professionals, and that the patient’s choices and preferences were respected, was important to the caregivers’ dignity.

My own dignity, ... I never really gave thought to it.... I am Fatima [fictitious name] and I come here for my dad. I come visit my dad and I hope he’ll be cared for well. And if not, I’ll get riled and then.... Some people were scared of me here. (#15)

For many caregivers, their dignity was also related to providing good care to the patient either by themselves or together with family members. Admission to a nursing home, in particular, was viewed by many as undignified. Even when such a care handover was considered necessary, some caregivers still felt they had “discarded” the patient, and that feeling affected their dignity.

Interviewer: And how has your own sense of dignity changed in this process? ... Or did you yourself feel or experience something in relation to this?

Respondent: In the beginning, I was very emotional, because we’d put her here [in the nursing home]. That we’d abandoned her here. Like, “Now you’re a burden. You’re a danger to yourself and your surroundings.” [But] what we’re doing is the best [option].... The responsibility that would come with it, I wouldn’t want it. Because of our history, I wouldn’t want it. But I’ll walk away from here. I’ll get in my car and go home. And I’ll leave her here. (#16)

Although informal caregivers put considerable effort into caring for their loved one, they sometimes felt others might not think it was enough effort. If others in the community did not acknowledge the amount of effort they put into the caregiving, or lacked knowledge about the disease and thus wrongly judged the amount of care needed, such views negatively influenced caregiver dignity.

Interviewer: Have you ever experienced anything in your surrounding [social circles] that affected dignity?

Respondent 1: That gossip by others, that will always happen.

Respondent 2: People can’t appreciate all the things you do. They try to belittle you. That makes you feel undignified. (#2, #3)

#### Dignified, despite giving up part of their own lives

Many informal caregivers described the impact of caring for their loved one in the last phase of life. The experience could be physically draining and emotionally tough; sometimes their lives had become ‘small’, at least temporarily. Being able to provide good care still remained important for the sake of their dignity.

You know, when I think about it—“Did I fall short in terms of care?”—I don’t think so. I did enough.... We did everything we could. Did you fall short? I think I had to give up a lot of my private life. What is dignity, then? You’re only caring and caring. You’re not thinking about where you are.... Yes, consciously you’re not really aware of those things. (#20)

Dividing the care and attention for the patient among family members was an effective way for informal caregivers to find a balance and continue providing care to the patient, and thereby maintain their own dignity.

#### Motives for the importance of family care

Informal caregivers cited several reasons why providing the care themselves was important to their own dignity. For example, they saw caregiving as part of maintaining a good relationship with the patient (see #10 below). Others emphasized that family care was important for the patient or the patient’s dignity (#8). Caregivers also stressed small but valuable aspects of family caregiving, such as having time for good conversations and reciting religious phrases for the patient (#8). For many Islamic caregivers, caring for family was also part of their religion. Surinamese caregivers did not mention a similar obligation; several also reported not being religious.

That we’re there for each other. I’m there for him. What if it was the other way around? Then he’d be there for me. So I don’t need any home care. I do it myself. (#10)

I said, “No, I don’t need [extra help]. I’ll take care of my husband myself.” Because my husband, psychologically—if an outsider were to come into our home—I thought he wouldn’t feel good about it, because my husband ... is a dignified man. [He would say,] “Someone else taking care of me—that’s a no-go.” (#8)

My kids always said, “Mom, that our dad was psychologically healthy was all thanks to you.” Because every night, I recited beautiful things for him. (#8)

Informal caregivers also pointed out that being cared for completely by family members was especially important to first-generation migrants (their own parents). That was part of informal caregivers’ motivation to provide the care themselves. They didn’t necessarily expect their children to do the same for them:

Actually, it’s not so terrible. I’d want to go to a nursing home if I were having a hard time, so as not make it too hard for my children. We think of it that way, but the first generation doesn’t see it like that. (#6)

### Dignity of the patient

In several ways, the dignity of the informal caregiver was related to the dignity of the patient. If a patient’s dignity was jeopardized by healthcare, the dignity of the caregiver was also affected. For example, one informal caregiver reported that being cared for by women rather than men was important to her mother’s dignity. When healthcare providers did not take her request seriously and she saw her mother being provided care by a man, her mother’s dignity suffered, as well as her own. In another interview, opinions of others in the community that the care being provided was not good enough damaged both the patient’s and the caregiver’s dignity simultaneously.

Caregiver dignity was influenced positively by dignified behavior on the part of the patient. For example, one caregiver reported that her husband’s dignified behavior involved having patience and surrendering to Allah’s will by praying. That dignified behavior reinforced her own caring role and made the role easier to carry out, which in turn was vital to her own dignity.

Interviewer: So you’re saying that his dignity, and his preservation of his dignity, comes from his faith?

Respondent: Yes, faith ... and his patience. I said to God, “O Lord, provide me with such patience. What is this patience, this patience?” Sometimes, at night, he couldn’t sleep, he just couldn’t sleep, and he didn’t even complain about why he couldn’t sleep. He just worshipped Allah then and prayed. And then he also prayed for me, so that also made it easy for me [to help him]. So it didn’t tire me out. (#8)

Another caregiver reported, however, that a patient’s lack of gratitude toward a healthcare professional negatively influenced her own dignity. When a care worker put extra effort into culturally personalized care, she would have considered it dignified behavior had the patient shown gratitude toward the care provider. The lack of such behavior made the informal caregiver feel undignified herself.

[The nurse] said, “I’ll cook Surinamese food with your mother. I hope she wants to help me....” But it was impossible to get [the patient] moving, [who said,] “No, I don’t feel like it, I’m not going.” Whereas that girl always puts effort into it.... And basically that makes me feel less dignified, because she just says, “No, I’m not going.” (#16)

### Interaction of informal caregivers with the patient and significant others

Several caregivers said their sense of dignity was interlinked with the nature of the relational interaction between them and the patient. Gratitude from the patient strengthened their dignity and their ability to provide care; aggressive behavior or thanklessness negatively affected their dignity.

The caregivers’ own behavior toward the patients also influenced their own sense of dignity. Two caregivers reported that becoming angry at the patient had negatively affected their dignity, if only temporarily, because they judged that as failure to provide the good care that was vital to their own dignity.

Interviewer: Was there also anything that was important for your own dignity?

Respondent: I had moments when I got very irritated—also toward my mother. [Like] one time when she argued with me, and then at one point I said, “Now you have to stop whining.” You know, that’s not kind of me either.... But it’s also good to put this into perspective afterwards—to say to yourself, you’re also just a human being and of course you’ll have quarrels sometimes. (#20)

Two caregivers also mentioned dignity in relation to significant others. When asked about their dignity, they explicitly noted that showing their emotions to others about the patient’s deterioration compromised their own dignity.

I didn’t want to show [my emotions] to my children. So, when they were here, I cried in the [other] room or after they left.... I also acted like everything was normal, in terms of both my tiredness [author’s note: she acted like she wasn’t tired] and the care for my husband. I didn’t show it to them and tried to not look sad and [did] like everything was perfect. (#8)

For the same caregivers, surrendering to Allah and providing care out of love seemed a major part of both their own and the patient’s dignity. This religious perspective itself appeared to contribute to their not wanting to show their emotions or complain about the situation, as they likened the behavior of “not complaining” to surrendering to Allah.

Interviewer: And how was your own dignity during that period?

Respondent: I was internally very peaceful. And he said a thousand times a day to me, “Oh, my dear, may Allah be pleased with you.” ... Then everything was fine for me.... He was sick, bedridden, for three to four years. I didn’t complain once.... I did it out of my own will, with love. (#8)

### Acknowledgment and support of the informal caretaking role by care professionals

Because providing care themselves and advocating for the patients’ needs and wishes is such a key element of caregiver dignity, it was also important for their dignity that healthcare professionals take informal caregivers seriously in that role and regard them as fully fledged partners.

Interviewer: In this whole process, is there anything that is important to your own dignity?

Respondent: I can see she’s not well and that things aren’t going as they should. [So it’s important to be] taken seriously as a designated representative of someone who can’t do or say anything about it herself. (#11)

Informal caregiver dignity appeared to be in particular jeopardy during decision-making phases at the end of life, such as about pain management or tube feeding, when differing opinions of physicians and informal caregivers with regard to such options may emerge. Informal caregivers wanted to be taken seriously in the sense that they could decide and choose what was best or most important for the patient. Caregivers viewed some responses of healthcare professionals as undignified, perceiving these as judgments about the value of the patient’s life or about the caregiver’s good intentions.

Every day they [the healthcare providers] would ask us again, “Do you want to think about it [the use of morphine]? She is in pain.” But she wasn’t, I really had the feeling she wasn’t suffering [from pain]. Of course, she had some breathing problems. But we were also there when my father died.... But the physician kept constantly saying, “Morphine, morphine, and stop the medication. Stop the tube feeding.” I thought, “Don’t you have anything else to say? We’ve already said ‘no’ several times. What are you doing in an Islamic unit? … Don’t you take me seriously as a legally designated representative? We didn’t just pick something. We really gave it thought together, as a family.” (#11)

Healthcare professionals could further preserve caregivers’ dignity by supporting their caring and advocating role. According to participants, such support includes their having complete information about the patient’s disease and prognoses, so they could know what awaits them and act accordingly for a dignified last phase of life for both patient and family. They also wished to be warmly treated when they visited or slept over. Caregivers also reported that they appreciated efforts by care professionals to support or explore a familial approach to caregiving, in which care for the patient would be shared among family members, in contrast to merely proposing a nursing home as a solution to the caregivers’ burden.

Interviewer: Was your own dignity affected during that period?

Respondent: I went to a psychologist.... They’re from another culture, you know. They say, “Let her live in a nursing home. Why are you doing this?” ... They don’t understand me.... After that, I went to a Turk.... They did a division of tasks. For example, my husband is in charge of the hospital stuff for my dad.... My daughter does the record-keeping.... In the beginning, I did all of that.... My son sleeps over three times a week. I thank him. (#6)

## Discussion

Prominent for the dignity of informal caregivers is their desire to ensure good care for the patient and preserve the patient’s dignity. To many respondents, that meant providing the patient care themselves; to others it meant advocating for good care and patient dignity in contacts with healthcare professionals and with other relatives. Even though caregivers reported that the physical and emotional impact of caregiving was heavy, being a good caregiver and ensuring good care from health professionals were vital to their own dignity. Many caregivers saw caregiving as part of maintaining a good relationship with their loved one and pointed out that providing care came with additional valuable aspects such as good conversations.

This study also highlighted the relational aspects of caregivers’ dignity. Their dignity was often linked to the patient’s dignity. Care that undermined the patient’s dignity also undermined their own dignity. Moreover, a patient’s behavior, or a caregiver’s own behavior toward the patient or significant others, could influence the caregiver’s own dignity. Caregivers’ dignity was strengthened when their role and their knowledge about the patient was acknowledged and supported by healthcare professionals.

To our knowledge, the dignity of informal caregivers has not previously been studied, and our finding that caregiver dignity is directly linked to good care for the patient and to the patient’s own dignity is therefore new. Caregivers provide care to a patient and, together with other relatives, they try to protect and strengthen the patient’s dignity. The relational interplay between patient and caregiver dignity is neglected in many models described in the current literature [3–6], which appear to focus solely on patient dignity. Our study has shown that many more aspects should be integrated into models about dignity. In particular that would involve an increased emphasis on the dignity of informal caregivers, including aspects such as their desire to provide the patient with good care and preserve patient dignity, as well as the relation between caregiver dignity and dignified behavior on the part of patients and informal caregivers. Dignity is shaped by those relationships [1].

Our findings could be specific to informal caregivers of migrant patients in a broader context. Caregivers in other countries who have comparable migrant backgrounds or similar religious views on caregiving might similarly view caring as a source of their own dignity. That may be especially true of persons from collectivistic countries; patients from our study in the Netherlands as well as those in the China and Taiwan studies were from collectivistic cultures [8, 9, 25]. However, generalization would be feasible only after other aspects such as health literacy, language barriers, and migration or integration into the host country have taken into account. Such factors could also influence matters like how caregivers view nursing home services and their own roles in providing care.

Given that providing care and, for some caregivers, not showing emotions about the situation have been shown to be major aspects of the dignity of informal caregivers, one might conclude that such caregivers neglect their own fatigue and support needs. Harding and Higginson indeed made such a finding, which they called a lack of self-identification, in their study of non-migrant European caregivers, and they concluded that informal caregivers operate between different pulling factors, such as care for their loved one and investing in their own future [26]. Our study showed, however, that caregivers of migrant patients did not feel they were operating between different pulling factors, even though they might neglect their own needs over a long period. They had their own motives for providing care, such as their relationship with the patient, the patient’s wish to take care of them, differences between generations, religious perspectives on caretaking, and opinions of others in the community. Hence, caring for loved ones and investing in one’s own future and needs are not necessarily seen as two opposing factors, but rather as interrelated concerns. Both researchers and care professionals should therefore be careful about negatively framing the low priority that caregivers give to their own needs while caring. That fails to do justice to the fact that caring for loved ones is a source of dignity for caregivers. Most of the current literature focuses on the burden of care among informal caregivers [27–29]. It is questionable whether that is the appropriate angle of support for informal caregivers in either migrant or native communities [30].

A much more beneficial way for care professionals to support migrant informal caregivers would be to communicate with them, keep them informed, involve them in decision-making, and acknowledge them in their caregiver role. Such insights are also found in other studies (focusing on other themes than dignity) among caregivers of non-migrant palliative patients, and they therefore seem relevant for all informal caregivers [31–33]. Our study noted that when healthcare professionals interacted with informal caregivers and patients whose values differed from their own, the professionals’ ability to safeguard caregiver and patient dignity acquired greater salience, and an insufficient response might foster distrust. If care providers imply that the decisions of informal caregivers may be damaging a patient’s dignity, whether justly or not, that may undermine the caregivers’ dignity and their caregiving role.

Healthcare professionals can support the dignity of informal caregivers of migrant patients by making sure to take them seriously in their caring role, by keeping them fully informed, by helping them build skills in providing good care, and by ensuring education about care and about the patient’s disease. Especially when serving migrant patients, care professionals could facilitate a familial approach to help family members share and manage the burden of care [34]. At the same time, they should be alert to feelings and emotions in informal caregivers as caregivers tend not to share these with others [34] as part of their dignity. Migrant caregivers could likewise be supported by welfare organizations that offer them opportunities to focus on exchanging experiences and building small-scale care skills [26], as well as providing emotional support to caregivers and discussing ways of caring for a loved one and maintaining dignity should a care handover to a nursing home be necessary. A further recommendation to healthcare professionals is to ensure respect for the perspectives and values of informal caregivers regarding a patient’s last phase of life when these reflect the patient’s wishes. Examples include issues of what constitutes quality of life, decisions on pain management and tube feeding, and the importance of religion in such decisions, particularly in cases where such values differ substantially from those of the professionals. If care professionals help to strengthen a patient’s dignity, that will also reinforce the dignity of the caregiver.

Although, as we have seen, delivering family care constitutes an integral part of dignity for many migrant caregivers, we want to stress that professional care providers should make sure to exclude or address certain other reasons why informal caregivers might insist on care given completely or largely by family, such as limited knowledge, negative images, or language barriers with regard to home care, or expectations that general practitioners will arrange it for them [15, 35]. Since migrant informal caregivers in other European countries may also regard giving care as a source of their dignity, these recommendations may also apply in other countries.

On a macro level, our findings imply that structural financial incentives could be made available to support informal caregivers who choose to take care of their loved ones [36]. Examples might include implementation of paid leave, reimbursement of transportation, provision of housing and other amenities for family members adjacent to care facilities, or even the availability of small payments for their informal care work, but further research is needed to determine what type of support would be most suiting [37–39]. Because many informal caregivers may eventually require extra help and some patients will need care in a nursing home, culturally competent care facilities and professionals, as well as quality professional care, will still be needed to preserve patient dignity, and hence the dignity of family caregivers.

A limitation of our study is that we had to interview across language barriers. The interviewers assured us, however, that they did have words for dignity and that people in their communities do talk about dignity. We also regularly discussed together other related words that aided in talking about dignity. By engaging well-trained, ethnically matched bilingual interviewers, we fostered trust among the participants and encouraged them to speak openly about their experiences and feelings [40]. Our findings could have been influenced by the fact that, in cases where a patient and caregiver were interviewed together, we used slightly concealing terms about the ‘current and future situation’ to inform participants about the focus of the interview—dignity in the last phase of life. Through this nondisclosure we believe we respected the wishes of patients and families, while still being able to obtain insights on the meaning of dignity. One further limitation is that the interviewed caregivers were predominantly female, because those were the primary informal caregivers or contact persons. We may have thereby missed important views of male relatives who were involved with the patient, as well as information on how the roles of various other relatives were interrelated.

## Conclusion

The dignity of informal caregivers of migrant patients in the last phase of life is closely entwined with ensuring good care for the patient and preserving the patient’s own dignity. Most research on dignity appears to focus solely on patient dignity. As a result, many models about dignity in the last phase of life fail to take into account the interaction between patients’ dignity and their caregivers’ dignity. Informal caregivers of migrant patients may neglect their own feelings with regard to the caregiving situation, but because taking care of the patient is an important source for their own dignity, an excessive focus on the caregivers’ burden of care may not be the right angle to address their feelings. Healthcare professionals could do more to strengthen the dignity of informal caregivers by supporting their role and showing respect for their values and perspectives regarding the last phase of the patient’s life.

## Supplementary Information

**Additional file 1.**

## Data Availability

Qualitative data used in this study cannot be shared due to reasons of confidentiality. Ethnic status is a personal characteristic that is confidential.

## References

[1] Leget C (2013). Analyzing dignity: a perspective from the ethics of care. Med Health Care Philos.

[2] Nordenfelt L (2004). The varieties of dignity. Health Care Anal.

[3] Van Gennip IE, Pasman HRW, Oosterveld-Vlug MG (2013). The development of a model of dignity in illness based on qualitative interviews with seriously ill patients. Int J Nurs Stud.

[4] Oosterveld-Vlug MG, Pasman HRW, Van Gennip IE (2014). Dignity and the factors that influence it according to nursing home residents: a qualitative interview study. J Adv Nurs.

[5] Choo PY, Ho GT, Dutta O, et al. Reciprocal dynamics of dignity end-of-life care: a multiperspective systematic review of qualitative and mixed methods research. AM J Hosp Palliat MeI. 2019:1–14. 10.1177/1049909119878860 31581779

[6] Chochinov HM, Hack T, McClement S (2002). Dignity in the terminally ill: a developing empirical model. Soc Sci Med.

[7] De Voogd X, Oosterveld-Vlug MG, Torensma M (2020). A dignified last phase of life for patients with a migration background: a qualitative study.

[8] Ho AHY, Chan CLW, Leung PPY (2013). Living and dying with dignity in Chinese society: perspectives of older palliative care patients in Hong Kong. Age Ageing.

[9] Li HC, Richardson A, Speck P (2014). Conceptualizations of dignity at the end of life: exploring theoretical and cultural congruence with dignity therapy. J Adv Nurs.

[10] WHO Definition of Palliative Care, https://www.who.int/cancer/palliative/definition/en/ (Accessed 29 Nov 2019).

[11] Van Gennip IE, Pasman HRW, Kaspers PJ (2013). Death with dignity form the perspective of the surviving family: a survey study among family caregivers of deceased older adults. Palliat Med.

[12] Oosterveld-Vlug MG, Pasman HRW, Van Gennip IE (2013). Nursing home staff’s views on residents’ dignity: a qualitative interview study. BMC Health Serv Res.

[13] Denktas S, Koopmans G, Birnie E (2009). Ethnic background and differences in health care use: a national cross-sectional study of native Dutch and immigrant elderly in the Netherlands. Int J Equity Health.

[14] Uiters E, Devillé W, Foets M, Groenewegen PP (2006). Use of health care services by ethnic minorities in the Netherlands: do patterns differ?. Eur J Pub Health.

[15] De Graaff FM, Francke AL (2003). Home care for terminally ill Turks and Moroccans and their families in the Netherlands: carers’ experiences and factors influencing ease of access and use of services. Int J Nurs Stud.

[16] De Graaff FM, Francke AL, Van den Muijsenbergh METC, et al. ‘Palliative care’: a contradiction in terms? A qualitative study of cancer patients with a Turkish or Moroccan background, their relatives and care providers. BMC Palliative Care. 2010;9(19). 10.1186/1472-684X-9-19 PMC2944252 20831777

[17] Centraal Bureau voor de Statistiek (CBS). Afbakening van generaties met migratieachtergrond, https://www.cbs.nl/nl-nl/achtergrond/2016/47/afbakening-generaties-met-migratieachtergrond (Accessed 9 Sept 2020).

[18] Ethikan I, Musa SA, Alkassim RS (2016). Comparison of convenience sampling and purposive sampling. Am J Theor Appl Stat.

[19] Murray SA, Kendall M, Boyd K (2005). Illness trajectories and palliative care. BMJ.

[20] Spencer L, Ritchie J, Huberman AM, Miles MB (2002). Qualitative data analysis for applied policy research. The qualitative Researcher's companion.

[21] Adams C (2017). Anders van Manen M. teaching phenomenological research and writing. Qual Health Res.

[22] Morse JM (2015). Critical analysis of strategies for determining rigor in qualitative inquiry. Qual Health Res.

[23] Central Committee on Research Involving Human Subjects. Your research: Is it subject to the WMO or not?, https://english.ccmo.nl/investigators/legal-framework-for-medical-scientific-research/your-research-is-it-subject-to-the-wmo-or-not (Accessed 27 Feb 2020).

[24] World Medical Association. WMA Declaration of Helsinki – Ethical principles for medical research involving human subjects. https://www.wma.net/policies-post/wma-declaration-of-helsinki-ethical-principles-for-medical-research-involving-human-subjects/ (Accessed 27 Feb 2020).

[25] De Valk H, Liefbroer AC, Esveldt I (2004). Family formation and cultural integration among migrants in the Netherlands. Genus.

[26] Harding R, Higginson I (2001). Working with ambivalence: informal caregivers of patients at the end of life. Support Care Cancer.

[27] Chiao CY, Wu HS, Hsiao CY (2015). Caregiver burden for informal caregivers of patients with dementia: a systematic review. Int Nurs Rev.

[28] Sawatzky JE, Fowler-Kerry S (2003). Impact of caregiving: listening to the voice of informal caregiver. J Psychiatr Ment Hlt.

[29] Goldstein NE, Concato J, Fried TR (2004). Factors associated with caregiver burden among caregivers of terminally ill patients with cancer. J Palliat Care.

[30] Grande G, Stajduhar K, Aoun S (2009). Supporting lay carers in end of life care: current gaps and future priorities. Palliat Med.

[31] Witkamp E, Droger M, Janssens R (2016). How to deal with relatives of patients dying in the hospital? Qualitative content analysis of relatives’ experiences. J Pain Symptom Manag.

[32] Linderholm M, Friedrichsen M (2010). A desire to be seen, family caregivers' experiences of their caring role in palliative home care. Cancer Nurs.

[33] Chambers M, Ryan AA, Connor SL (2001). Exploring the emotional support needs and coping strategies of family carers. J Psychiatr Ment Hlt.

[34] Ahmad M, Van den Broeke J, Saharso S, et al. Persons with a migration background caring for a family member with dementia: challenges to shared care. Gerontologist. 2019:1–10. 10.1093/geront/gnz161 PMC7039377 31786594

[35] Suurmond J, Rosenmöller DL, El Masbahi H (2015). Barriers in access to home care services among ethnic minority and Dutch elderly – a qualitative study. Int J Nurs Stud.

[36] Keefe J, Rajnovich B (2007). To pay or not to pay: examining underlying principles in the debate on financial support for family caregivers. Can J Aging.

[37] Lai DWL (2012). Effect of financial costs on caregiving burden of family caregivers of older adults. Health Psychol.

[38] Gardiner C, Taylor B, Robinson J, Gott M (2019). Comparison of financial support for family caregivers of people at the end of life across six countries: a descriptive study. Palliat Med.

[39] Greenfield JC, Hasche L, Bell LM, Johnson H (2018). Exploring how workplace and social policies relate to caregivers’ financial strain. J Gerontol Soc Work.

[40] Lee SK, Sulaiman-Hill CR, Thompson C (2014). Overcoming language barriers in community-based research with refugee and migrant populations: options for using bilingual workers. BMC Int Health Hum Rights.

