# Supplementary material for: Dignity of informal caregivers of migrant patients in the last phase of life: a qualitative study
Source: BMC Palliat Care. 2021 Feb 4;20:26. doi: 10.1186/s12904-021-00721-6 (PMC7863486; doi:10.1186/s12904-021-00721-6)
Supplement: Supplementary file 1 — Additional file 1. [file 12904_2021_721_MOESM1_ESM.doc]

# Appendix 1

# Interview informal caregiver

**Introduction and preparation**

I will be asking you questions about what is important for maintenance of the dignity of your loved one. This can be questions that might be hard for you or can make you emotional or sad. We ask these questions because we want care to be in line with wishes and perspectives of patients. There are no right or wrong answers. We are glad to hear everything that you want to tell us.

EXPLORING THE SITUATION

**Can you tell me something about the patient’s illness and his/her situation?**

*Think about: illness, disorder, duration of the illness up to now, experience of the illness, symptoms, informal caregiver’s thoughts about what might come, involved others (professionals, informal caregivers and relatives), in what way the informal caregiver is involved with care for the patient.*

Example questions

Which illness does your loved one have?

When did he/she get this diagnosis?

What is he/she able to de him or herself and what not, as a consequence of the illness?

Which care or medical examinations will you have in the future?

What type of care do you have / receive?

Who are involved? (professionals, other relatives)

What is your role in this?

DIGNITY PATIENT

**What do you think is or is not dignified for your loved one in the context of his or her illness?**

Explanation: As a consequence of being ill, some things might have changed for him or her. What kind of influence do these changes have on feeling dignified?

*For example: less physical activity, becoming dependent, less privacy*

**What influences feeling dignified?**

- Probe on positive and negative influences
- Probe: What are solutions for not feeling dignified?

**What do you think is important for maintenance of a sense of dignity of your loved one?**

INFLUENCE OF RELATIONSHIPS WITH OTHERS

**What do you think is important to do yourself to preserve the dignity of your loved one?**

- What do you need for that?

**What role do others have to preserve the dignity of your loved one?**

*Think about: family, acquaintances or the community.*

**What can care professionals contribute to or infringe the dignity of the patient?**

*Think about: practically and psychosocially and several care professionals such as physicians, nurses and other caregivers*

DIGNITY OF THE INFORMAL CAREGIVER

**How do you experience your own dignity?**

Probe on negative and positive experiences.

**What contributes to preserving your own dignity?**

**What can other contribute to preserving your own dignity?** (family, friends and acquaintances**)**

**How could care contribute to your dignity?**

**How could care professionals contribute to your dignity?**

THE FUTURE

I will now ask you a couple of questions about the last days of life. Maybe you have thought about before or maybe you experienced the last phase of life of someone close to you. I would like to ask you some questions about what would be important in these last days.

**Do you sometimes think about the future and what would be important for your loved one? (or do you worry about that sometimes?)**

- What do you expect? *Think about receiving care, place of caregiving, who would be involved)*

**Did you ever experience the last phase of life of a loved one?**

**Did you ever think about how the last days with your loved one would be?**

*Think about the importance of rituals, food and drinks and religion)*

- Did you think about this together?
- Does your loved one has specific wishes?
- Do you have specific wishes?
- Probe on what would be important for their own dignity and that of their loved one.
- What does a physician need to know about your religion or culture?

Are you involved with decision making about treatments or medical examinations? Who do you want to be involved with decision making?

*Think about different kind of settings: patient only, patient and family, physician and patient together, physician and patient and family.*

FINAL QUESTIONS

**What are your values based on or where do they originate from?**

*Think about religion, culture of country of birth, family, parents, former experiences with the last phase of life*

**Did we address everything? Have you got something to add or ask?**

PERSONAL DATA

How old are you? And your loved one?

Which education have you got? Which education does your loved one have?

*This might had been abroad.*

- *Also ask for the level of education: primary school, secondary school, intermediate professionals education, higher professional education or university.*

What is / was your occupation? What was your loved one’s occupation?

Where are you born? Where is your loved one born?

What is your religion? What is the religion of your loved one?

Where do you live? Where does your loved one live?

Which disease(s) does your loved one have? (Which disease did the physician tell you that he or she has got?)

Which care does your loved one uses?

Care environment (at home, nursing home etc.):

Gender:

*Position of the interviewee*

**Which cultural group do you feel you belong to? And your loved one?**

**Do you have specific reasons for that?**
